# A Comparative In Vivo Scrutiny of Biosynthesized Copper and Zinc Oxide Nanoparticles by Intraperitoneal and Intravenous Administration Routes in Rats

**DOI:** 10.1186/s11671-018-2497-2

**Published:** 2018-04-03

**Authors:** Ashajyothi C, Harish K Handral, Chandrakanth Kelmani R

**Affiliations:** 1 Medical Biotechnology and Phage Therapy Laboratory, Department of Post Graduate Studies and Research in Biotechnology, Gulbarga University, Gulbarga, Karnataka 585 106 India; 2 Department of Biotechnology, Maharajah’s Post Graduate College, Vizianagaram, Andhra Pradesh India; 3 Medicine and Physiology Department, University Health Network, Toronto, Canada

**Keywords:** In vivo study, Biogenic copper nanoparticles, Biogenic zinc oxide nanoparticles, Intraperitoneal route, Intravenous route, Serum creatinine

## Abstract

During the present time, anti-microbial features of copper (Cu) and zinc oxide (ZnO) nanoparticles (NPs) are extensively used to combat the growth of pathogenic microbes. CuNPs and ZnONPs are recurrently used in cosmetics, medicine and food additives, and their potential for toxic impacts on human and ecosystem is of high concern. In this study, the fate and toxicity of 16- to 96-nm-ranged biosynthesized copper (Bio-CuNPs) and zinc oxide (Bio-ZnONPs) was assessed in male Wistar rats. In vivo exposures of the two nanoparticles are achieved through two different administration routes namely, intraperitoneal (i/p) and intravenous (i/v) injections. The three different concentrations, no observable adverse effect concentration (NOAEC), inhibitory concentration (IC_50_) and total lethal concentration (TLC), were appraised at the dose range of 6.1 to 19.82 μg/kg and 11.14 to 30.3 μg/kg for Bio-CuNPs and Bio-ZnONPs respectively, for both i/p and i/v routes on 14th and 28th day of observation. These dose ranges are considered based on the previous study of antibacterial dose on multidrug-resistant pathogenic bacteria. In this study, we investigated the toxic effect of Bio-CuNPs and Bio-ZnONPs on animal behaviour, animal mass, haematologic indices, organ indices and histopathology of liver, spleen, kidney and brain organs. We found that i/v and i/p administration of Bio-ZnONPs in three different doses did not cause mortality and body weight was slightly reduced up to second week of administration compared with the vehicle control group. At the dose ranges of 11–16 μg/kg (i/v) and 24–30 μg/kg (i/p), no significant changes were observed in the serum creatinine level as well as serum ALT, serum AST level and ALP level which were 40.7 mg/dl, 37.9 IU/L and 82.4 IU/L normal as compared to vehicle control on 14th and 28th day of observation. These findings are confirmed in liver, kidney and spleen indices and histopathology studies. Furthermore, liver and kidney injury occurred when the concentrations of Bio-CuNPs were at 9.5 μg/kg (IC_50_) and 11.7 μg/kg (TLC) for i/v route of administration. Similarly, increase in serum ALT (67.7 mg/dl), AST level (70 IU/L) and ALP (128 IU/L) was also observed. And the body weight was significantly lower than in the control group after 14th day, and there were statistically significant differences observed by this route; interestingly, the toxicity of Bio-CuNPs in serum is prolonged (up to 28th day). Effect of Bio-CuNPs through i/p route was considerably low as compare to the control. Results of the present study revealed that Bio-ZnONPs have no effect on kidney and liver function biomarkers (both i/v and i/p) as compared to Bio-CuNPs.

**Electronic supplementary material:**

The online version of this article (10.1186/s11671-018-2497-2) contains supplementary material, which is available to authorized users.

**Graphical abstract:**
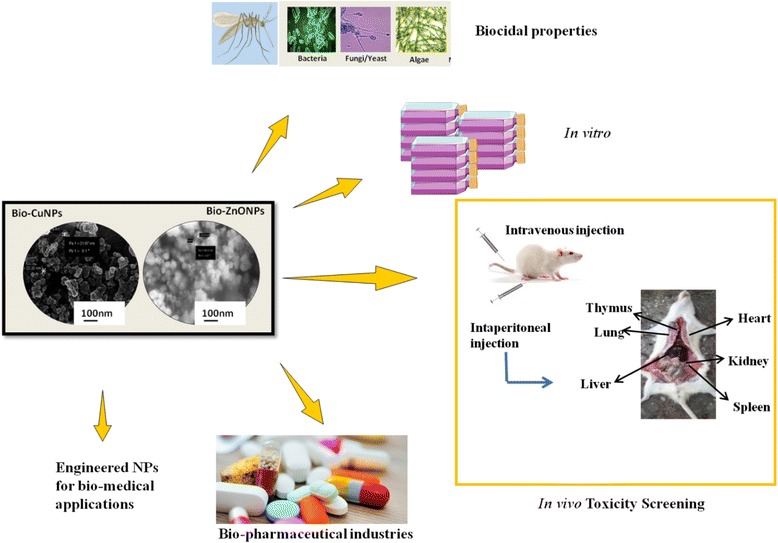
As shown in graphical abstract (Fig. 1), our aim is to assess the toxicity of Bio-CuNPs and Bio-ZnONPs through in vivo protocol. According to Kahru and Dubourguier reviews, AgNPs, CuNPs and ZnONPs have been historically used as biocides, for preventing the growth of microorganisms and algae (Kahru and Dubourguier 2010). Therefore, as like pesticides, nanomaterials should be monitored for their toxic response toward non-target species, including humans and animals. To gain a better understanding whether the accidental release of metal-containing NPs may pose a threat to non-target species, assessing of toxic effect is indispensable.The ‘non-target organism’ is an organism which will be exposed to NPs after their incidental release into the environment.

## Background

Certain metals are required for the normal physiological functions in living organisms. Since the past decade, there has been increase in the use of metal based NPs in bio-medical applications, exponential use of NPs alerts the safety concerns to reduce and/or prevent NP induced adverse effects on the living system [1]. Among NPs, Cu and ZnO are generally found in the food supplements and human body [2, 3]. Unique physiochemical properties of Cu and ZnONPs attain functional applications in physiological metabolic processes, thus increasing their commercial value in industries [4–6]. However, adverse effects, including hemolysis, gastrointestinal distress, and liver and kidney damage were observed upon excess intake of Cu and ZnONPs [7].

Particularly, the absorption of CuNPs is readily takes place after the ingestion, inhalation and the dermal exposure [8, 9], significantly through the gastrointestinal tract [8, 10]. CuNPs target mucosal cells and retain inside by bonding with metallothionein or glutathione [11]. It is stored primarily in the liver, brain, heart, kidney and muscles. It was reported, 98% of Cu binds with Ceruloplasmin, a serum protein which lead to cellular toxicity. [12, 13].Cu is a catalytic inducer of superoxide radicals, hydroxyl radicals and hydrogen peroxide via the Haber-Weiss reaction [14], higher concentrations of Cu may cause oxidative induced stress.

Based on the extent of solubility ZnONPs were being considered as separate group of NPs within the metal oxide NPs [15]. Zinc element is found in human body and ZnONPs are known to be of lower toxic [3]. However, excessive zinc is reported to induce toxic effects [16]. Release of metallic cations Zn^2^from ZnONPs are also proved to be toxic in micro-organisms and rodents [17]. ZnO NPs might enter via different routes to reach blood flow and induce adverse impacts on organs [18]. Preliminary results indicated ZnONPs affected organ systems may show inflammation, altered heart rate and functions, and oxidative stress [19, 20]. According to [21], inhalation of 20 nm ZnONPs (2.5 mg/kg bw) by rats twice a day resulted in an increased Zn content in the liver after 12 h and in kidneys after 36 h.

Increased awareness towards nanotoxicity, studies have been reported on the in vivo toxicity of CuNPs and ZnONPs for intranasal instillation [22, 23], intratracheal instillation [24, 25] and oral administration [26–28], dermal exposure [29, 30]. In order to evaluate the toxicity intravenous (i/v) and intraperitoneal (i/p) administration needs has to be performed. To our knowledge, minimal reports are available on CuNPs and ZnONPs toxicity for intravenous and intraperitoneal administration. Moreover, the toxicological mechanism and tissue distribution of the two NPs have not yet been systematically studied on following i/v and i/p injection.

Hereby, we demonstrated the toxicity of 16-96 nm ranged biosynthesized CuNPs and ZnONPs in male wistar rats through intraperitoneal (i/p) and intravenous (i/v) injectionsat the desired on 14th and 28th day of observation.

## Method

### Biosynthesis of bio-CuNPs and bio-ZnONPs

Biological synthesis of CuNPs and ZnONPs from non-pathogenic *Enterococcus faecalis* was adapted by extracellular enzymatic method [31, 32]. Further, the shape and size of synthesized nanoparticles were confirmed using field emission scanning electron microscopy (FeSEM) and transmission electron microscopy (TEM).

### In vivo studies

#### Experimental animals and animal husbandry

Specific disease-free, 12- to 13-week-old male Wistar rats were purchased from Mahaveera Enterprises, Hyderabad, India. Animals were selected within a weight range of 160–200 g for each group and acclimatized for 1 week prior to the initiation of treatment, and health status of rats was monitored daily. The animals were housed under standard conditions of temperature (24 ± 1 °C) and relative humidity (55 ± 10%) respectively, in 12-h light/dark cycles. During treatment, animals were housed in cages with stainless steel mesh lids. Animals were fed with commercially available standard pellet diet (VRK Nutrition Solutions, Sangli, Maharashtra, India Ltd.). Drinking water was supplied to the animals, ad libitum.

Toxicity studies were performed at Luqman College of Pharmacy, Kalaburagi, India. Animal handling was performed according to Good Laboratory Practice. The study protocol was approved by Institute Animals Ethics Committee (Approval number: 346/CPCSEA).

#### Preparation and Administration of Bio-Cu and ZnONPs

The stock suspensions of Bio-CuNPs and Bio-ZnONPs (50 mg/ml) were prepared by dissolving separately in double distilled water for overnight and were filtered using 0.22 μ syringe filters. Filtrates are used to prepare the working standard ranging from 1.25-175 μg/ml concentration.

Animals were divided into three groups of three different concentrations for each nanoparticle type. Considering six rats/group for intravenous route (coded as experiment A set) and six rats/group for intraperitoneal route (coded as experiment B set), as per Tables 1 and 2. In both experiment sets *group A* served as control (vehicle distilled water).

**Table 1:** Intravenous route of administration of three different concentrations for each Biogenic nanoparticles: experiment (A)

Group II: Bio-CuNP (μg/kg)	Group III: Bio-ZnONPs (μg/kg)
Group B (NOAEC) 6.1	Group B (NOAEC) 11.14
Group C (IC_50_) 9.5	Group C (IC_50_) 13.7
Group D (TLC) 11.7	Group D (TLC) 16. 21

**Table 2:** Intraperitoneal route of administration of three different concentrations for each Biogenic nanoparticles: experiment (B)

Group II: Bio-CuNP (μg/kg)	Group III: Bio-ZnONPs (μg/kg)
Group B (NOAEC) 13.41	Group B (NOAEC) 24.8
Group C (IC_50_) 16.75	Group C (IC_50_) 27.2
Group D (TLC) 19.82	Group D (TLC) 30.3

*NOAEC* “no observable adverse effect concentration”, *IC*_*50*_ inhibitory concentration, *TLC* total lethal concentration

#### Observation and examination items

##### Clinical signs

During the test, post treatment observation was done once in a day to monitor the signs of clinical toxicity and/or death.

##### Feed and water consumption

Feed and water consumption was recorded daily after the starting date of treatment, was calculated from the differences between the supplied amounts and the remaining amounts.

##### Animal behaviour and body weight

Every two days after injection, rats were weighed and assessed for behavioral changes.

#### Hematological indices

Using a standard saphenous vein blood collection technique, blood was drawn for hematology analysis (using potassium-methylenediaminetetraacetic acid collection tubes). According to the standard hematologic analysis 300 μl of blood was collected from the rat and on 14 and 28 days standard hematologic parameters, i.e, platelet count, hematocrit, hemoglobin, red blood cell count, and white blood cell count were analyzed [33].

#### Biochemistry panel analysis of serum

To determine the serum biochemical levels including the alanine aminotransferase (ALT/GPT), creatinine (CRE), aspartate aminotransferase (AST) and alkaline phosphatase (ALP), control and treated rats were sacrificed and whole blood samples were collected for centrifugation (3000 rpm) for 15mins. Assessment was performed by an automatic biochemical analyzer for 14 and 28th day’s samples [34].

#### Detection of organelles weight

After 14 and 28 days, the rats were anesthetized by ether with phosphate-buffered saline and were dissected. Organs of the control and treated groups were harvested immediately. Heart, lung, thymus, brain, kidney, liver and spleen were separated carefully and washed with sodium chloride solution and rinsed with ice-cold deionized water and dried with filter paper. Morphology and color of dissected organs were investigated and weight of each organ was measured. To examine the grade of changes explicitly caused by Bio-Cu and ZnONPs, the Organ index (O_*X*_) was calculated separately by using formula [35]:

$$ \mathrm{Organ}\ \mathrm{index}\ \left({\mathrm{O}}_{\mathrm{X}}\right)=\frac{\mathrm{Weight}\ \mathrm{of}\ \mathrm{experimental}\ \mathrm{organ}/\mathrm{weight}\ \mathrm{of}\ \mathrm{experimental}\ \mathrm{animal}}{\mathrm{Weight}\ \mathrm{of}\ \mathrm{control}\ \mathrm{organ}/\mathrm{weight}\ \mathrm{of}\ \mathrm{control}\ \mathrm{animal}} $$

Where Organ Index (O_X_) may change as:

Heart index (H_X_), Liver index (Li_X_), Spleen index (S_X_), Lung index (Lu_X_), Kidney index (K_X_), Thymus index (T_X_), Brain index (B_X_).

#### Histology

One rat from each group including control was fixed with 10% buffered formalin following phosphate-buffered saline exsanguinations. A small piece of liver, kidney, spleen and brain was fixed by 10% formalin and embedded into paraffin. Paraffin blocks were sectioned and processed for hematoxylin and eosin staining. Stained sections were observed using bright field microscopy [36].

#### Statistical analysis

All data are expressed in mean ± SD of the mean of the three independent experiments; each was carried out in triplicate, *N* = 6 rats per group.

## Results and Discussion

Synthesis of Bio-CuNPs and Bio-ZnONPs were carried out by extracellular enzymatic method during exposure of reactants to *Enterococcus faecalis* supernatant. FeSEM analysis of Bio-CuNPs and Bio-ZnONPs can be seen with size ranging from 1 to 100 nm in distribution (Additional file 1). TEM analysis reports the presence of biosynthesized CuNPs and ZnONPs with core shell morphology of size 12–90 nm and spherical in shape for CuNPs [31] and ZnONPs ranging from 16 to 96 nm [32] (Additional file 2).

Evaluation of Bio-Cu and ZnONPs on male wistar rats was investigated with no sign of mortality upon NP treatment. Furthermore, after the treatment and till the end of experiment duration frequent examination of white feces was monitored after i/v administration of Bio-CuNPs at 9.5 to 11.5 μg/kg dosage. Between third and fourth week, Bio-CuNPs’ treated rats have showed significant rise in feed and water consumption for i/v was 9.5 μg/kg (IC_50_) and 11.5 μg/kg (TLC) whereas for i/p route(dose range: 24.8 to 30.3 μg/kg) and control group rats from 3rd to 4th week.Variation in the body weight of rats after i/v and i/p administration of Bio-CuNPs and Bio-ZnONPs was shown in Tables 3 and 4.Reduction and increase in the body weight are valuable indicators in assessing the toxicity of a test sample [37]. Previous reports evidenced in toxicity studies on 13.5 nm gold [33] and 100 nm silver [38] NPs effect on body weight by i/v injection was less than i/p and oral administration. According Rhiouani et al.*,* the low decrease in weight after 4 days of treatment in all of the treated groups can suggest adverse effects of toxic substances on the animals [39].

**Table 3:** Body weight changes for rat treated with Bio-CuNPs and Bio-ZnONPs through intravenous route of administration

Days after injection	Group I: Bio-CuNPs’ body weight (g)			Group II: Bio-ZnONPs’ body weight (g)			Control
	NOAEC	IC_50_	TLC	NOAEC	IC_50_	TLC	Body weight (g)
1	100 ± 7.74	100 ± 6.65	100 ± 6.43	100 ± 3.75	100 ± 6.89	100 ± 4.83	100 ± 5.56
2	100 ± 4.43	100 ± 5.43	98.5 ± 5.73	100 ± 05.73	100 ± 5.54	100.5 ± 4.93	100 ± 6.45
4	105.4 ± 5.76	102 ± 7.75	96.2 ± 4.93	102 ± 4.0	106.2 ± 5.78	102.4 ± 5.93	106 ± 5.51
6	108.3 ± 6.92	104 ± 3.32	96 ± 5.93	103.6 ± 5.84	106.5 ± 4.93	104.8 ± 5.81	105 ± 5.37
8	96.5 ± 3.84	102 ± 6.84	94.8 ± 4.75	108 ± 5.83	105.1 ± 5.21	104.8 ± 6.83	108 ± 6.48
10	95.3 ± 4.73	100 ± 5.93	90.6 ± 3.94	108.2 ± 6.75	106.8 ± 4.83	104.6 ± 4.83	112 ± 4.76
12	95 ± 5.84	100 ± 4.99	88.6 ± 2.4	104.2 ± 5.93	106.6 ± 5.99	104.8 ± 6.93	116 ± 5.6
14	95.3 ± 4.05	98.5 ± 5.32	88 ± 3.2	109.2 ± 4.9	104.5 ± 6.084	105.25 ± 4.83	116 ± 6.71
16	98.6 ± 4.74	96.4 ± 4.95	84.2 ± 4.095	109.5 ± 6.55	102.7 ± 6.032	105.8 ± 5.897	121 ± 7.56
18	98 ± 5.84	92.3 ± 3.99	80.8 ± 3.83	109.8 ± 6.4	102 ± 7.083	106.2 ± 7.54	121 ± 8.53
20	96.5 ± 4.75	90.7 ± 4.73	80 ± 3.73	110.7 ± 5.83	103.5 ± 6.65	104.8 ± 5.95	121 ± 5.42
22	98 ± 3.83	88.5 ± 3.93	75.5 ± 4.94	114.6 ± 6.93	104.8 ± 4.4	103.7 ± 6.84	121 ± 6.34
24	98 ± 6.84	88 ± 2.73	73.7 ± 4.93	115.8 ± 6.43	107.7 ± 5.32	102.5 ± 5.93	124 ± 4.3
26	95.6 ± 4.95	82.3 ± 4.75	70.4 ± 4.04	117.2 ± 4.23	108.45 ± 6.83	100.02 ± 3.65	124 ± 5.82
28	95.5 ± 3.95	80.9 ± 3.65	68.9 ± 3.93	119.5 ± 5.73	110.7 ± 4.6	102.65 ± 3.84	124 ± 6.568

All data are expressed in mean ± SD of the mean of the three independent experiments; each was carried out in triplicate, *N* = 6 rats per group

**Table 4:** Body weight changes for rat treated with Bio-CuNPs and Bio-ZnONPs through intraperitoneal route of administration

Days after injection	Group I: Bio-CuNPs’ body weight (g)			Group II: Bio-ZnONPs’ body weight (g)			Control
	NOAEC	IC_50_	TLC	NOAEC	IC_50_	TLC	Body weight (g)
1	100 ± 5.2	100 ± 6.73	100 ± 8.3	100 ± 5.21	100 ± 5.26	100 ± 6.94	100 ± 5.23
2	100.2 ± 6.63	100 ± 7.99	100.5 ± 5.28	100.4 ± .6.54	100.25 ± 6.5	100 ± 6.32	102 ± 6.7
4	99.7 ± 6.94	102.6 ± 7.073	99.4 ± 7.83	101.7 ± 6.66	100.7 ± 6.3	99.8 ± 4.21	102 ± 7.84
6	98.4 ± 7.73	102.3 ± 9.093	99.2 ± 7.94	101.8 ± 7.21	99.7 ± 4.1	99.56 ± 3.75	105 ± 8.8
8	99.7 ± 5.45	100.2 ± 8.702	97.3 ± 5.45	102.2 ± 5.64	99.6 ± 3.93	98.6 ± 4.03	106 ± 7.32
10	100.8 ± 4.84	99.3 ± 5.88	97.6 ± 6.35	104.8 ± 8.21	100.67 ± 8.5	97.23 ± 3.2	106 ± 5.46
12	102.7 ± 6.33	99.3 ± 6.93	96.2 ± 7.44	106.2 ± 6.94	100.8 ± 7.3	96.8 ± 5.3	108 ± 6.94
14	100 ± 7.21	98 ± 7.3	96.3 ± 5.95	106.7 ± 7.74	98.4 ± 4.6	94.5 ± 6.86	108 ± 6.42
16	102 ± 6.42	101.5 ± 6.45	97.7 ± 6.83	107 ± 6.2	97.43 ± 3.4	96.6 ± 4.31	110 ± 5.67
18	104 ± 4.73	102.7 ± 8.32	98.3 ± 5.79	108.5 ± 8.05	98.7 ± 5.5	98.5 ± 5.94	110 ± 7.12
20	106 ± 5.88	104 ± 5.6	98.6 ± 4.67	110.2 ± 7.34	97.2 ± 5.3	96.7 ± 4.7	112 ± 5.09
22	105.4 ± 6.063	102.4 ± 9.5	99.2 ± 3.45	112.5 ± 6.21	100.3 ± 6.74	99.6 ± 4.43	114 ± 6.56
24	106.7 ± 7.83	103.8 ± 5.6	100 ± 5.23	112.6 ± 5.92	102.6 ± 7.2	99.7 ± 3.78	118 ± 7.58
26	107.3 ± 6.08	103 ± 5.7	100.5 ± .6.05	114.5 ± 7.12	103.23 ± 6.78	100.5 ± 5.34	119 ± 4.53
28	109.4 ± 5.73	105.7 ± 7.6	102.9 ± 6.85	117.8 ± 8.49	104.45 ± 5.54	99.8 ± 4.95	120 ± 6.25

All data are expressed in mean ± SD of the mean of the three independent experiments; each was carried out in triplicate, *N* = 6 rats per group

**Fig. 1:**
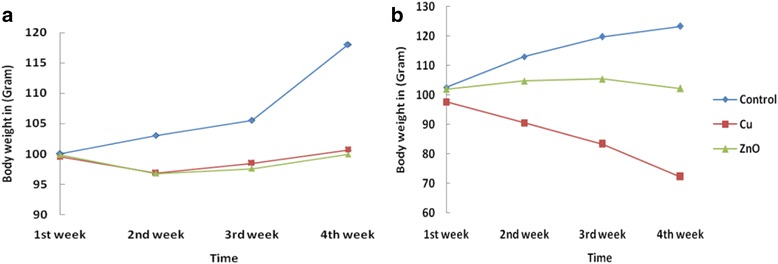
Change in body weight of untreated (control) and treated rats with Bio-CuNPs and Bio-ZnONPs. Bio-CuNPs and Bio-ZnONPs treated through (A) intraperitoneal (i/p) and (B) intravenous (i/v) route of administration up to 28th day of observation. All treatments of Bio-CuNPs (Cu) and Bio-ZnONPs (ZnO) at their total lethal concentration were administered and kept in observation for 28 days; *N* = 6 rats per group

It can be seen that i/v and i/p administration of Bio-ZnONPs in the three different doses (NOAEC, IC_50_ and TLC) body weight was slightly reduced up to second week of administration compared with the control group. However, after 14th day body weight was regained. In case of i/p administration, reduction in body weight was induced by Bio-ZnONPs (30.3 μg/kg) at total lethal concentration and was lower than control group, thus indicating trivial toxicity via i/p route over the i/v route(Table 4, Fig. 1a). Similarly, rats treated with Bio-CuNPs, at 9.5 μg/kg and 11.7 μg/kg concentration via i/v route slight reduction in the body weight was noticed. Till 14 days treatment with Bio-CuNPs no sign adverse effects on growth and body weight gain were observed. Body weight variation within 28 days at a dose of 11.7 μg/kg (i/v route) is shown in Table 3. After 14th day of treatment, it was found considerable decrease in body weight via i/v route when compared with the control group. Thus, indicates toxicity of Bio-CuNPs via this route (Fig. 1b). Bio-CuNPs treated rats via i/p route administration induced minor decrease in body weight and no sign of mortality was observed in both i/p and i/v routes. Therefore, i/p injections induced lower toxicity (shown in Table 4 and Fig. 1a).

### Hematology indices

Estimation of hematologic parameters such as, RBC count, WBC count, platelet count, hemoglobin level and blood clotting time are the important entities to measure the toxicity of treated NPs. For day 14 and 28, concentration-dependent hematology results arepresented in Tables 5 and 6 for i/p and i/v route of administration. Dosage of Bio-CuNPs at 9.5 μg/kg (IC_50_) and 11.7 μg/kg (TLC) via i/v route has shown reduced RBC count in contrast to Bio-ZnONPs. However, concentration-dependent trend has not been noticed. For rats treated with Bio-ZnONPs through i/v route of administration, hemoglobin level, platelet count, and white blood cells have changed, but no significant difference is observed among all three concentrations (NOAEC, IC_50_ and TLC). But in case of i/p route of administration, significant decrease and changes in red blood cell count, white blood cells, hemoglobin level and platelet count has been found on 14th day of observation (Table 5) as compared to the control and Bio-CuNPs treated. Surprisingly, hematologic effects are found to be normal on 28th day (Table 6).

**Table 5:** Haematology study for rat treated with Bio-CuNPs and Bio-ZnONPs through intravenous and intraperitoneal route of administration after 14th day of observation

Parameters	Group I: Bio-CuNPs			Group II: Bio-ZnONPs			Control	Ref. range
	NOAEC	IC_50_	TLC	NOAEC	IC_50_	TLC		
WBC								
i/v	10.4 ± 0.76	2.46 ± 0.015	11.94 ± 0.53	10.45 ± 0.53	10.8 ± 0.69	9.7 ± 0.82	9.71 ± 0.98	4–10
i/p	9.7 ± 0.64	3.08 ± 0.053	12.5 ± 0.629	10.1 ± 0.72	9.38 ± 0.79	10.84 ± 0.443	9.82 ± 0.094	
RBC								
i/v	4.51 ± 0.043	2.82 ± 0.081	1.2 ± 0.07	5.81 ± 0.38	4.95 ± 0.58	5.23 ± 0.64	4.98 ± 0.072	3.50–5.50
i/p	5.174 ± 0.7	4.05 ± 0.039	5.94 ± 0.043	5.56 ± 0.053	4.65 ± 0.013	3.12 ± 0.32	4.67 ± 0.053	
Hb								
i/v	129 ± 5.83	92 ± 3.6	115 ± 5.86	152 ± 5.7	152 ± 5.39	158 ± 4.3	127 ± 5.32	110–160
i/p	103.64 ± 6.9	100 ± 3.88	144 ± 3.64	131 ± 4.5	142 ± 3.92	160 ± 6.69	132 ± 4.054	
Platelet Count								
i/v	45 ± 4.53	21 ± 2.5	435 ± 6.3	276 ± 6.2	253 ± 6.7	238 ± 5.8	232 ± 5.3	100–300
i/p	208 ± 6.43	106.2 ± 4.2	55.8 ± 3.96	280 ± 5.8	310 ± 7.5	322 ± 6.65	245 ± 4.5	
Clotting time								
i/v	7.2 ± 0.0065	8.4 ± 0.005	0.7 ± 0.0083	5.2 ± 0.0017	4.8 ± 0.0063	4.72 ± 0.0032	6.6 ± 0.012	2–5
i/p	4.2 ± 0.0017	1.8 ± 0.0015	7.5 ± 0.0064	5.7 ± 0.0074	5.3 ± 0.0041	5.5 ± 0.0083	5.9 ± 0.005	

All data are expressed in mean ± SD of the mean of the three independent experiments; each was carried out in triplicate, *N* = 6 rats per group

*i/v* intravenous route, *i/p* intraperitoneal route, *NOAEC* no observable adverse effect concentration, *IC*_*50*_ inhibitory concentration, *TLC* total lethal concentration, *WBC* white blood cells (SI unit 10^9^/L), *RBC* red blood cells (SI unit 10^9^/L), *Hb* haemoglobin (SI unit g/L) and platelet count (SI unit 10^9^/L), clotting time: in minutes

**Table 6:** Haematology study for rat treated with Bio-CuNPs and Bio-ZnONPs through intravenous and intraperitoneal route of administration after 28th day of observation

Parameters	Group I: Bio-CuNPs			Group II: Bio-ZnONPs			Control	Ref. range
	NOAEC	IC_50_	TLC	NOAEC	IC_50_	TLC		
WBC								
i/v	12.4 ± 0.089	3.8 ± 0.039	10.6 ± 0.005	9.5 ± 0.069	9.7 ± 0.0089	9.93 ± 0.072	7.67 ± 0.043	4–10
i/p	8.2 ± 0.062	7.6 ± 0.057	9.8 ± 0.017	10 ± 0.074	9.45 ± 0.083	10.2 ± 0.037	7.23 ± 0.032	
RBC								
i/v	5.6 ± 0.82	3.82 ± 0.081	2.2 ± 0.07	4.8 ± 0.027	5.5 ± 0.0083	5.18 ± 0.0032	5.2 ± 0.005	3.50–5.50
i/p	4.174 ± 0.033	4.6 ± 0.086	6.6 ± 0.079	5.18 ± 0.0071	5.57 ± 0.065	6.2 ± 0.084	4.94 ± 0.027	
Hb								
i/v	30.7 ± 2.7	98.5 ± 4.72	120 ± 6.52	158 ± 4.57	154.6 ± 5.4	150 ± 5.83	134.5 ± 5.76	110–160
i/p	130.1 ± 4.83	134.7 ± 6.79	142.4 ± 6.4	143.7 ± 5.63	145.8 ± 3.89	154.4 ± 7.65	137.8 ± 4.93	
Platelet Count								
i/v	52 ± 2.83	34.9 ± 3.6	356.8 ± 5.93	280 ± 2.83	284 ± 5.45	256.9 ± 4.29	247.2 ± 7.52	100–300
i/p	273.8 ± 4.95	258.2 ± 5.72	73.9 ± 3.44	284 ± 5.57	288.9 ± 6.93	294.3 ± 6.076	278.4 ± 6.06	
Clotting time								
i/v	7 ± 0.94	8.3 ± 0.74	0.89 ± 0.64	4.92 ± 0.93	4.9 ± 0.6	4.7 ± 0.043	5.5 ± 0.06	2–5
i/p	4.5 ± 0.25	1.5 ± 0.73	7.2 ± 0.86	5 ± 0.64	5.17 ± 0.09	5 ± 0.085	5.6 ± 0.03	

All data are expressed in mean ± SD of the mean of the three independent experiments; each was carried out in triplicate, *N* = 6 rats per group

*i/v* intravenous route, *i/p* intraperitoneal route, *NOAEC* no observable adverse effect concentration, *IC*_*50*_ inhibitory concentration, *TLC* total lethal concentration, *WBC* white blood cells (SI unit 10^9^/L), *RBC* red blood cells (SI unit 10^9/^L), *Hb* haemoglobin (SI unit g/L) and platelet count (SI unit 10^9^/L), clotting time: in minutes

The hematologic effects of the different injection methods (i/v, i/p) for the two different Bio-NPs on 14th and 28th day of observations are diverse. It can be observed that hemoglobin, red blood cells, white blood cells and platelets decrease via i/v route in Bio-CuNPs treatment and via i/p route in Bio-ZnONPs treated rats. But the significant decrease in RBC counts was observed. This indicates that the different injection routes did not induce significant differences in platelet count, hemoglobin, or white blood cells except in rats injected with Bio-CuNPs (i/v route). Red blood cells show a significant difference after i/p and i/v injection (as shown in Tables 5 and 6).

### Biochemical assay of serum

**Fig. 2:**
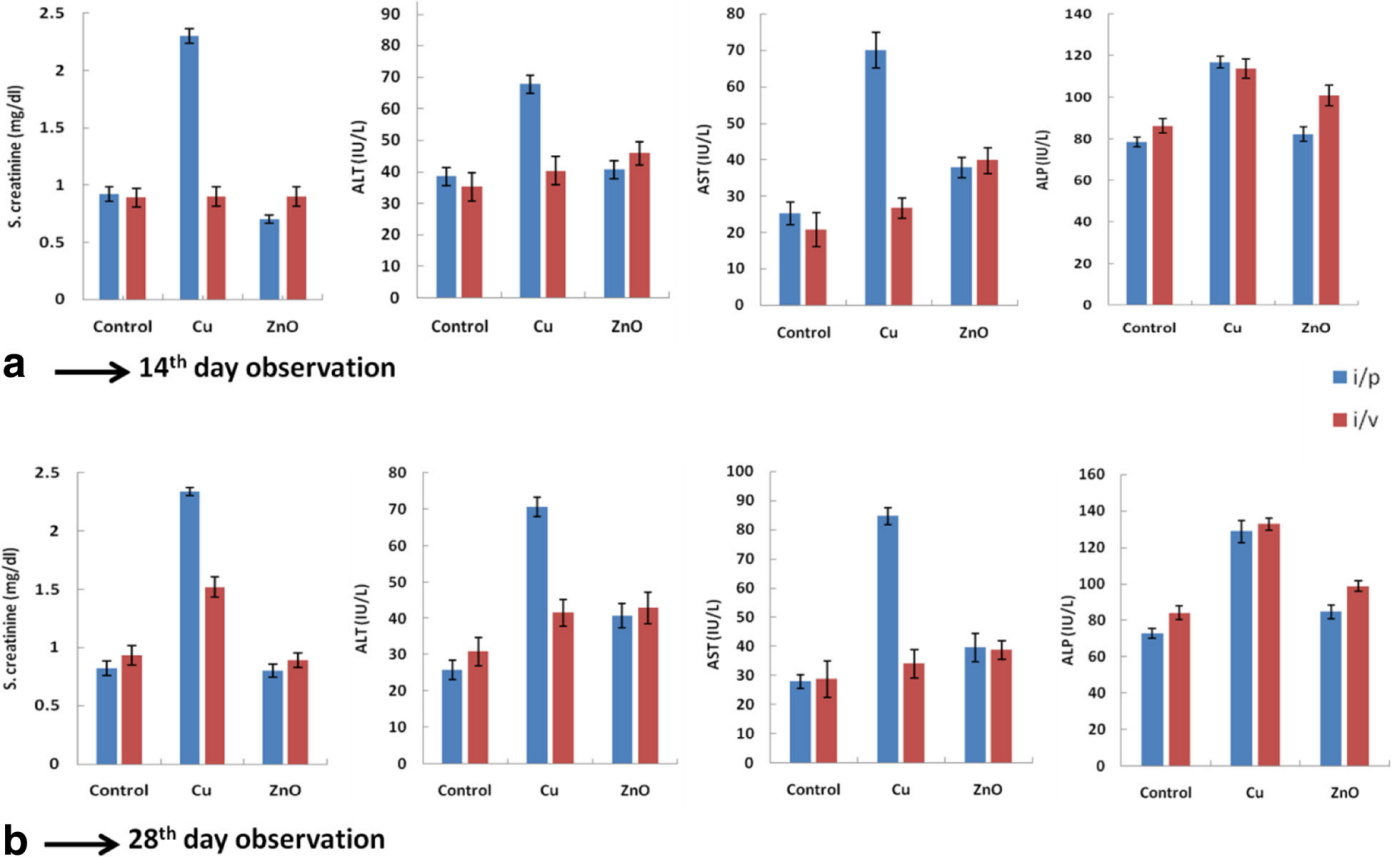
Biochemical results of rats treated with Bio-CuNPs and Bio-ZnONPs. S. creatinine, ALT, AST and ALP levels were measured in rats treated with Bio-CuNPs and Bio-ZnONPs through intraperitoneal (i/p) and intravenous (i/v) route of administration on (A) 14th day and (B) 28th day. All data are expressed in mean ± SD of the mean of the three independent experiments; each was carried out in triplicate, *N* = 6 rats per group. Note: Cu: Bio-CuNPs, ZnO: Bio-ZnONPs, i/p: intraperitoneal, i/v: intravenous

Serum creatinine is waste product; higher creatinine production indicates kidney damage. Bio-ZnONPs (i/v route: dosage of 11-16 μg/kg, i/p route: dosage of 24-30 μg/kg) not significantly affected serum creatinine level when compared with control on 14th and 28th day. (Tables 7 and 8, Fig. 2a, b). Rats treated with Bio-CuNPs (i/v route: dosage of 06-12 μg/kg) have showed increase in serum creatinine level to 2.3 mg/dl when compared with control. However,i/p route of injection did not showed significant changes (Tables 7 and 8). The blood serum has large number of enzymes but to assess the normal and pathological symptoms of liver, alanine transaminse (glutamate pyruvate transaminase) and aspartate transaminases (glutamate oxalate acetate transaminase) are useful. Aspartate transaminase is of mitochondrial origin present in large quantities in liver, heart, kidney and skeletal muscles. Serum alkaline phosphatase is a globulin enzyme of low molecular weight, found in higher concentration in bones, hepatobiliary tract and kidney. The activity of this enzyme can be determined by the estimation of organic phosphate liberated from the glycerol phosphate. The serum level of the enzymes was increased in both hepatocellular and obstructive Jaundice. In the i/v route of administration, Bio-ZnONPs (40.7 mg/dl, 37.9 IU/L, 82.4 IU/L) no significanteffects on serum ALT, serum AST and ALP levels as compared with control. Although i/p administration showed significant increase in ALT, AST and ALP level as compared with control at 14th and 28th day (Fig. 2a and b). Results of toxicity study on serum showed Bio-ZnONPs no changes in the levels of creatinine, ALT, AST and ALP levels for i/v route till 28 days.

**Table 7:** Biochemical assays for rat treated with Bio-CuNPs and Bio-ZnONPs through intraperitoneal and intravenous route of administration for 14th day of observation

Tests	Group I: Bio-CuNPs			Group II: Bio-ZnONPs			Control	Ref. range
	NOAEC	IC_50_	TLC	NOAEC	IC_50_	TLC		
S. creatinine								
i/v	0.9 ± 0.083	1.5 ± 0.067	2.3 ± 0.035	0.7 ± 0.043	0.6 ± 0.075	0.78 ± 0.074	0.92 ± 0.023	0.7–1.4
i/p	0.72 ± 0.076	0.75 ± 0.087	0.9 ± 0.023	0.83 ± 0.068	0.93 ± 0.074	0.9 ± 0.073	0.89 ± 0.056	
ALT								
i/v	35.2 ± 3.75	44.6 ± 2.74	67.7 ± 3.84	12.7 ± 4.64	32.8 ± 3.54	40.7 ± 4.32	38.5 ± 4.85	0.0–40.0
i/p	30.8 ± 2.56	35.7 ± 4.45	40.3 ± 2.75	17.8 ± 3.92	20.8 ± 4.73	45.9 ± 2.43	35.2 ± 3.15	
AST								
i/v	27.3 ± 2.65	30.8 ± 3.77	70 ± 2.78	24.6 ± 3.67	30.7 ± 2.43	37.9 ± 3.51	25.2 ± 3.18	5.0–34.0
i/p	20.8 ± 3.23	24.9 ± 4.78	26.7 ± 3.56	17.4 ± 1.93	20.8 ± 2.84	39.8 ± 2.57	20.7 ± 4.72	
ALP								
i/v	75.8 ± 4.43	84.8 ± 2.85	116.8 ± 3.38	75.8 ± 2.89	78.9 ± 3.17	82.4 ± 3.85	78.5 ± 2.38	37–103
i/p	83.9 ± 3.68	98.3 ± 3.43	113.7 ± 4.85	72.8 ± 3.64	80.8 ± 2.94	100.8 ± 4.93	86.3 ± 3.5	

All data are expressed in mean ± SD of the mean of the three independent experiments; each was carried out in triplicate, *N* = 6 rats per group

*i/v* intravenous route, *i/p* intraperitoneal route, *NOAEC* no observable adverse effect concentration, *IC*_*50*_ inhibitory concentration, *TLC* total lethal concentration, S. creatinine (mg/dl), *ALT* alanine aminotransferase (IU/L), *AST* aspartate aminotransferase (IU/L), *ALP* alkaline phosphatase (IU/L)

**Table 8:** Biochemical assays for rat treated with Bio-CuNPs and Bio-ZnONPs through intraperitoneal and intravenous route of administration for 28th day of observation

Tests	Group I: Bio-CuNPs			Group II: Bio-ZnONPs			Control	Ref. range
	NOAEC	IC_50_	TLC	NOAEC	IC_50_	TLC		
S. creatinine								
i/v	0.95 ± 0.073	1.7 ± 0.057	2.34 ± 0.035	0.7 ± 0.061	0.72 ± 0.059	0.8 ± 0.058	0.82 ± 0.062	0.7–1.4
i/p	0.9 ± 0.038	1.48 ± 0.093	1.52 ± 0.085	0.8 ± 0.079	0.92 ± 0.093	0.89 ± 0.062	0.93 ± 0.083	
ALT								
i/v	37.5 ± 1.82	46.8 ± 1.44	70.6 ± 2.73	22.1 ± 2.81	34.2 ± 3.98	40.7 ± 3.32	25.8 ± 2.64	0.0–40.0
i/p	35.8 ± 2.032	37.8 ± 3.93	41.6 ± 3.64	20.9 ± 0.067	22.8 ± 2.74	42.9 ± 4.32	30.9 ± 3.93	
AST								
i/v	28.2 ± 3.82	36.5 ± 5.16	84.7 ± 2.9	27.8 ± 5.44	32.7 ± 4.84	39.5 ± 4.8	27.8 ± 2.29	5.0–34.0
i/p	26.3 ± 3.83	28.2 ± 2.17	33.9 ± 4.82	28.3 ± .3.77	30.8 ± 3.51	38.7 ± 3.2	28.7 ± 0.063	
ALP								
i/v	73.7 ± 3.38	90.8 ± 2.63	128.7 ± 5.93	75.3 ± 3.83	80.6 ± 3.79	84.7 ± 3.7	72.8 ± 2.75	37–103
i/p	84.2 ± 4.84	100.2 ± 5.79	132.7 ± 3.36	74.7 ± 2.88	84.8 ± 4.68	98.8 ± 2.82	84.2 ± 3.68	

All data are expressed in mean ± SD of the mean of the three independent experiments; each was carried out in triplicate, *N* = 6 rats per group

*i/v* intravenous route, *i/p* intraperitoneal route, *NOAEC* no observable adverse effect concentration, *IC*_*50*_ inhibitory concentration, *TLC* total lethal concentration, S. Creatinine (mg/dl), *ALT* alanine aminotransferase (IU/L), *AST* aspartate aminotransferase (IU/L), *ALP* alkaline phosphatase (IU/L)

In contrast, rats treated with Bio-CuNPs through i/v route, showed significant increase in serum ALT (67.7 mg/dl), AST level (70 IU/L), and ALP (128 IU/L). Effect of Bio-CuNPs through i/p route was considerably low compared to control. The difference of the results could be attributed to difference in the dosage routes, toxicity of nanoparticles as well as the duration of administration. We found Bio-ZnONPs have no effect on kidney and liver function biomarkers (both i/v and i/p) as compare to Bio-CuNPs.

### Detection of organelles weight and histological study

Changes in the organ weights of rat at different Bio-NPs doses, illustrate the adverse effects of NPs on organs. It can be seen that the weights of the heart, liver, spleen, lung, kidneys, and brain are decreased in rats when treated with Bio-ZnONPs as depicted in Tables 9 and 10. Furthermore, consideration of the organ reaction and grade of changes were examined by calculating organ index (O_X_) of each organ separately. The organ index for heart, liver, spleen, lung, kidneys, brain and thymus are presented in Tables 9 and 10.

**Table 9:** Weight of organs or organ index (O_X_) for rat treated with Bio-CuNPs and Bio-ZnONPs through intraperitoneal and intravenous route of administration for 14th day of observation

Tests	Group I: Bio-CuNPs			Group II: Bio-ZnONPs			Control
	NOAEC	IC_50_	TLC	NOAEC	IC_50_	TLC	
Heart (H_X_)							
i/v	0.96 ± 0.06	0.48 ± 0.07	0.22 ± 0.075	1.1 ± 0.04	1.03 ± 0.065	1.25 ± 0.024	1 ± 0.02
i/p	1.9 ± 0.09	1.25 ± 0.0344	1.143 ± 0.023	1.23 ± 0.032	0.98 ± 0.077	0.94 ± 0.065	1.4 ± 0.05
Liver (Li_X_)							
i/v	I.25 ± 0.043	0.92 ± 0.06	0.48 ± 0.049	1.67 ± 0.026	1.56 ± 0.093	1.47 ± 0.086	1.8 ± 0.043
i/p	1.44 ± 0.05	1.23 ± 0.08	1.08 ± 0.099	1.54 ± 0.07	1.34 ± 0.032	1.27 ± 0.0289	1.67 ± 0.032
Spleen (S_X_)							
i/v	0.59 ± 0.07	0.47 ± 0.09	0.265 ± 0.043	1.45 ± 0.019	0.97 ± 0.006	0.87 ± 0.054	1.16 ± 0.028
i/p	087 ± 0.01	0.63 ± 0.054	0.58 ± 0.052	1.38 ± 0.054	0.66 ± 0.053	0.65 ± 0.039	1.18 ± 0.067
Lungs (Lu_X_)							
i/v	0.87 ± 0.065	0.77 ± 0.076	0.42 ± 0.081	2.14 ± 0.098	.78 ± 0.033	1.54 ± 0.044	1.56 ± 0.082
i/p	0.86 ± 0.086	0.67 ± 0.012	0.75 ± 0.093	2.06 ± 0.12	0.94 ± 0.054	0.78 ± 0.067	1.72 ± 0.028
Kidneys (K_X_)							
i/v	0.64 ± 0.03	0.5 ± 0.043	0.35 ± 0.073	2.4 ± 0.156	2.10 ± 0.028	1.78 ± 0.026	1.23 ± 0.099
i/p	0.83 ± 0.06	0.8 ± 0.053	0.65 ± 0.043	1.98 ± 0.087	0.34 ± 0.054	0.88 ± 0.058	1.52 ± 0.091
Thymus (T_X_)							
i/v	0.21 ± 0.09	0.18 ± 0.027	0.11 ± 0.053	0.167 ± 0.034	0.15 ± 0.034	0.142 ± 0.094	0.18 ± 0.076
i/p	0.2 ± 0.03	0.22 ± 0.036	0.18 ± 0.09	0.178 ± 0.053	0.112 ± 0.028	0.13 ± 0.027	0.16 ± 0.045
Brain (B_X_)							
i/v	1.92 ± 0.05	0.72 ± 0.072	0.75 ± 0.001	2.3 ± 0.044	1.76 ± 0.095	1.54 ± 0.079	1.89 ± 0.092
i/p	1.76 ± 0.086	1.25 ± 0.058	1.22 ± 0.064	2.1 ± 0.075	1.21 ± 0.053	1.02 ± 0.091	1.72 ± 0.087

All data are expressed in mean ± SD of the mean of the three independent experiments; each was carried out in triplicate, *N* = 6 rats per group

**Table 10:** Weight of organs or organ index (O_X_) for rat treated with Bio-CuNPs and Bio-ZnONPs through intraperitoneal and intravenous route of administration for 28th day of observation

Tests	Group I: Bio-CuNPs			Group II: Bio-ZnONPs			Control
	NOAEC	IC_50_	TLC	NOAEC	IC_50_	TLC	
Heart (H_X_)							
i/v	0.94 ± 0.003	0.8 ± 0.0021	0.67 ± 0.003	1.5 ± 0.0056	1.24 ± 0.0048	1.45 ± 0.0087	1.4 ± 0.094
i/p	1.69 ± 0.005	1.65 ± 0.0067	1.43 ± 0.004	1.45 ± 0.0078	1.23 ± 0.0054	1.14 ± 0.0095	1.32 ± 0.037
Liver (Li_X_)							
i/v	1.27 ± 0.0053	0.99 ± 0.0032	0.67 ± 0.0043	1.77 ± 0.0064	1.66 ± 0.005	1.59 ± 0.0078	1.8 ± 0.005
i/p	1.48 ± 0.083	1.32 ± 0.008	1.18 ± 0.0027	1.69 ± 0.0043	1.45 ± 0.0952	1.2 ± 0.0074	1.78 ± 0.090
Spleen (S_X_)							
i/v	0.67 ± 0.0045	0.53 ± 0.053	0.49 ± 0.004	1.35 ± 0.0067	1.23 ± 0.0034	1.16 ± 0.0043	1.2 ± 0.004
i/p	1.87 ± 0.067	1.63 ± 0.003	1.52 ± 0.0087	1.58 ± 0.054	1.56 ± 0.0034	1.04 ± 0.0037	1.8 ± 0.0058
Lungs (Lu_X_)							
i/v	1.4 ± 0.083	1.56 ± 0.0056	1.18 ± 0.0067	2.1 ± 0.0065	1.99 ± 0.0065	1.58 ± 0.0056	1.59 ± 0.0043
i/p	1.86 ± 0.006	1.61 ± 0.0078	1.24 ± 0.0064	2.17 ± 0.031	1.94 ± 0.0043	1.58 ± 0.0074	1.68 ± 0.005
Kidneys (K_X_)							
i/v	1.64 ± 0.085	1.2 ± 0.056	1.13 ± 0.0067	2.3 ± 0.063	2.52 ± 0.0083	2.17 ± 0.0067	1.7 ± 0.0093
i/p	1.63 ± 0.093	1.23 ± 0.0067	0.95 ± 0.0084	2.67 ± 0.0034	1.78 ± 0.0089	0.98 ± 0.0052	1.6 ± 0.0031
Thymus (T_X_)							
i/v	0.158 ± 0.004	0.124 ± 0.066	0.11 ± 0.0043	0.164 ± 0.0053	0.16 ± 0.0042	0.157 ± 0.0034	0.16 ± 0.0073
i/p	0.21 ± 0.0052	0.23 ± 0.0082	0.21 ± 0.0065	0.175 ± 0.0098	0.169 ± 0.0063	0.15 ± 0.0089	0.174 ± 0.0065
Brain (B_X_)							
i/v	1.92 ± 0.0-76	0.78 ± 0.0067	0.76 ± 0.0056	2.18 ± 0.0064	1.97 ± 0.0078	1.85 ± 0.0037	1.92 ± 0.045
i/p	1.92 ± 0.054	1.67 ± 0.0032	1.32 ± 0.0053	2.6 ± 0.0056	1.92 ± 0.0053	1.73 ± 0.0063	1.85 ± 0.0074

All data are expressed in mean ± SD of the mean of the three independent experiments; each was carried out in triplicate, *N* = 6 rats per group

**Fig. 3:**
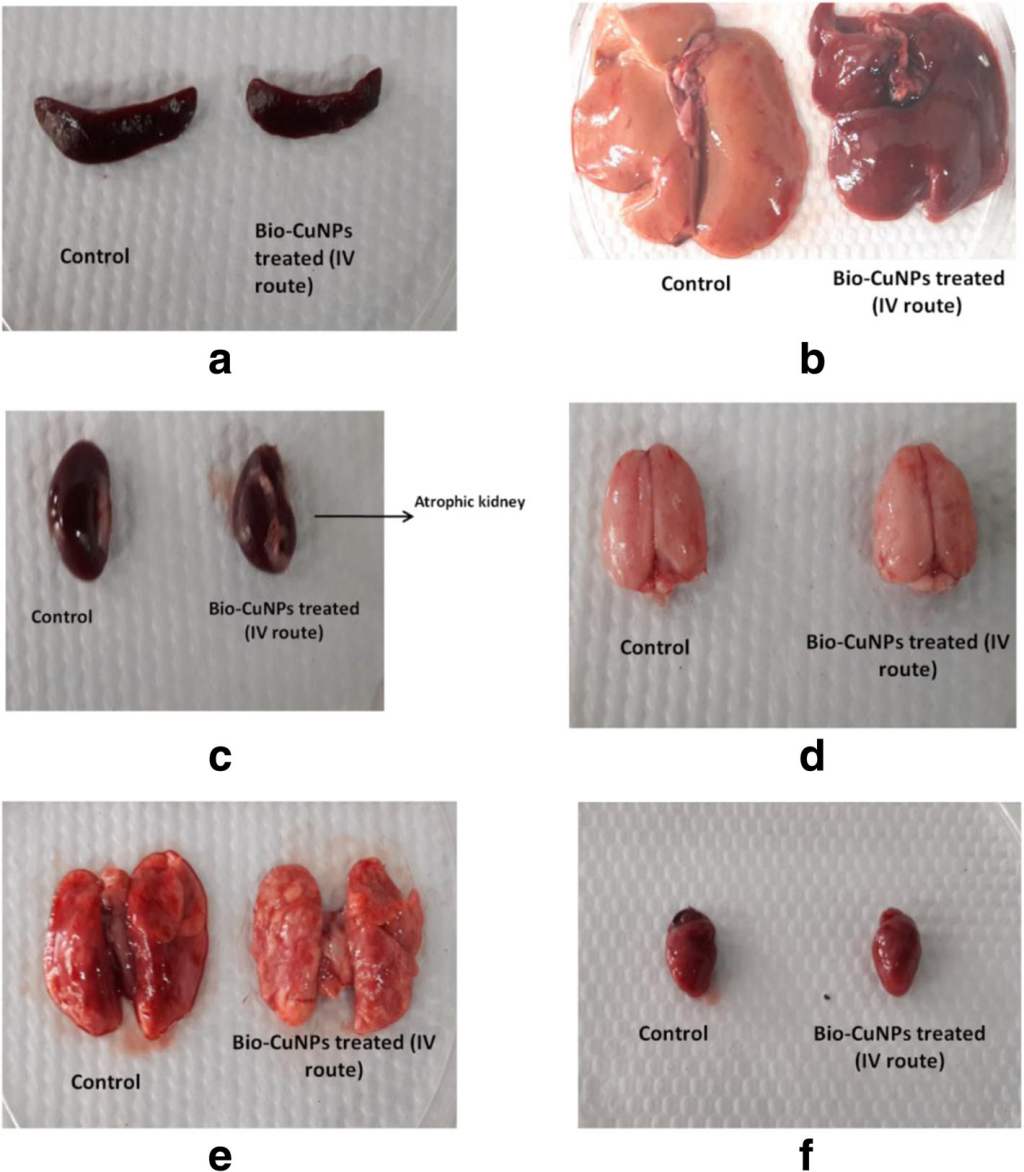
Change in organelle morphology. Where **a** spleen, **b** liver, **c** kidney, **d** brain, **e** lungs and **f** heart, of male Wistar rats treated with Bio-CuNPs via intravenous route in comparison with control on 28th day observation

Difference in the weight of spleen and thymus were observed after i/v and i/p administration in Bio-CuNPs and Bio-ZnONPs treated rats. On 14th day, Bio-ZnONPs has shown decreased spleen index via i/p injection, and increased by i/v administration (Table 9). In case of Bio-CuNPs treated rats via i/v administration showed significant reduction in spleen index on 14th (0.265) and 28th day (0.49). Thus indicating, the immune system has been affected by i/v Bio-CuNPs administration and i/p Bio-ZnONPs administration. In case of, i/p Bio-ZnONPs administration, immune system of rat is recurring to the normal state after 14th day and proves the effect is not prolonged. Taken together with the previous body weight variation, it seems that i/v administration route of Bio-CuNPs can affect the heart, liver, lung, kidneys, and brain; furthermore it might damage the immune system. From Fig. 3a, it implies spleen and thymus are main target of organs by Bio-CuNPs.

In case of i/v and i/p Bio-CuNPs treated groups obvious effects on organ index has been observed at both IC_50_ and TLC doses. Moreover, of the two different administration routes, the intraperitoneal injection show the modest toxicity in Bio-ZnONPs treated groups and highest toxicity in Bio-CuNPs treated groups. Efficient drug absorption by i/p injection was known to be rapid due to the dense blood vessels and lymph in the murine peritoneum [40]. Correspondingly, the intravenous injection shows the least toxicity in Bio-ZnONPs treated groups and highest toxicity in Bio-CuNPs treated groups.

### Toxicological changes in rats

**Fig. 4:**
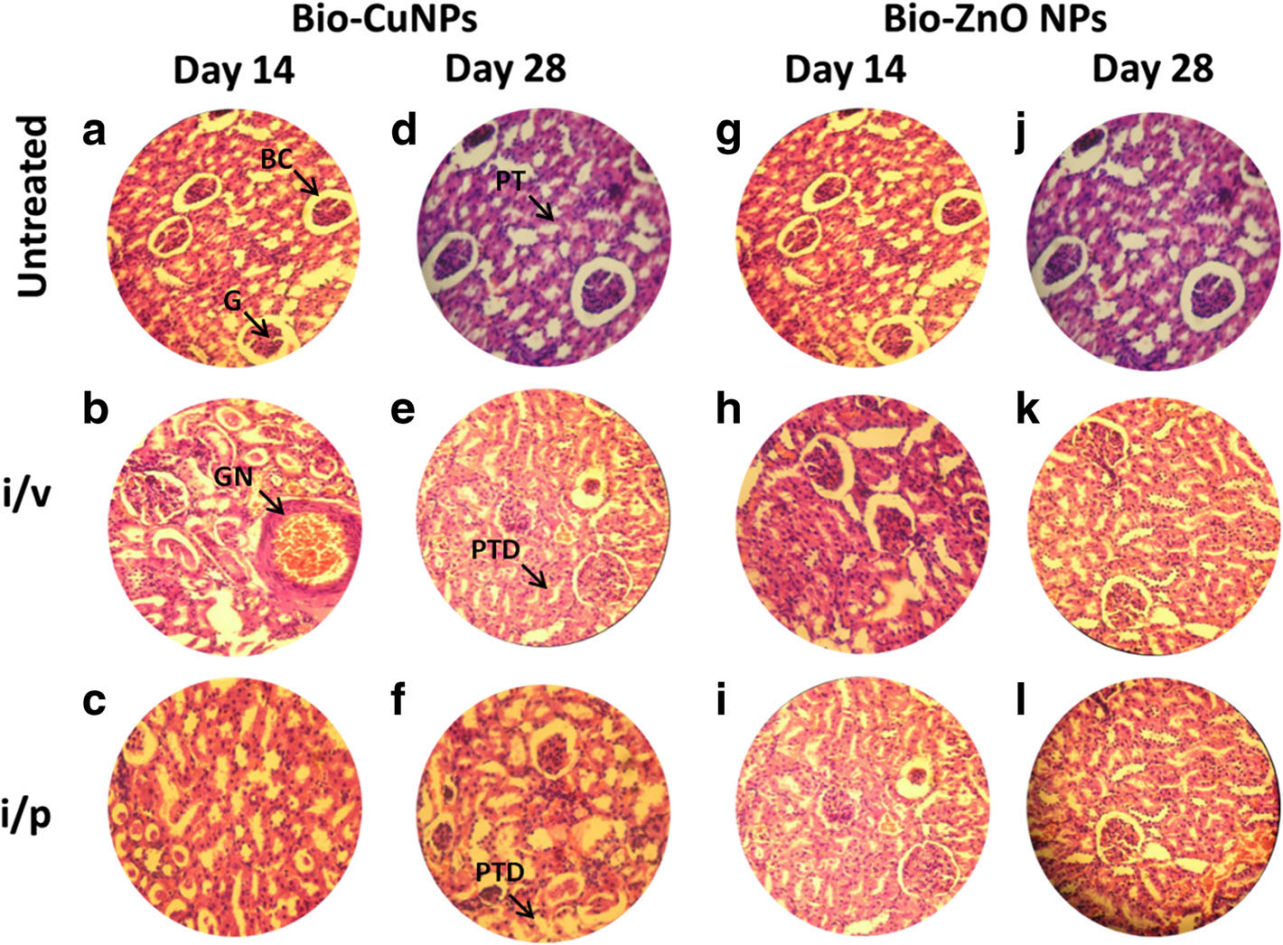
H&E-stained sections of rat kidney. Rats treated via i/v and i/p route with Bio-Cu and ZnONPs; untreated samples were considered as control. Where sections treated with Bio-CuNPs observed on day 14 (A–C) and day 28 (D–F). Bio-ZnONPs’ treated sections on day 14 (G–I) and day 28 (J–L). BC Bowman capsule, G glomerular, PT proximal tubule, GN glomerular necrosis, PTD proximal tubule damage

**Fig. 5:**
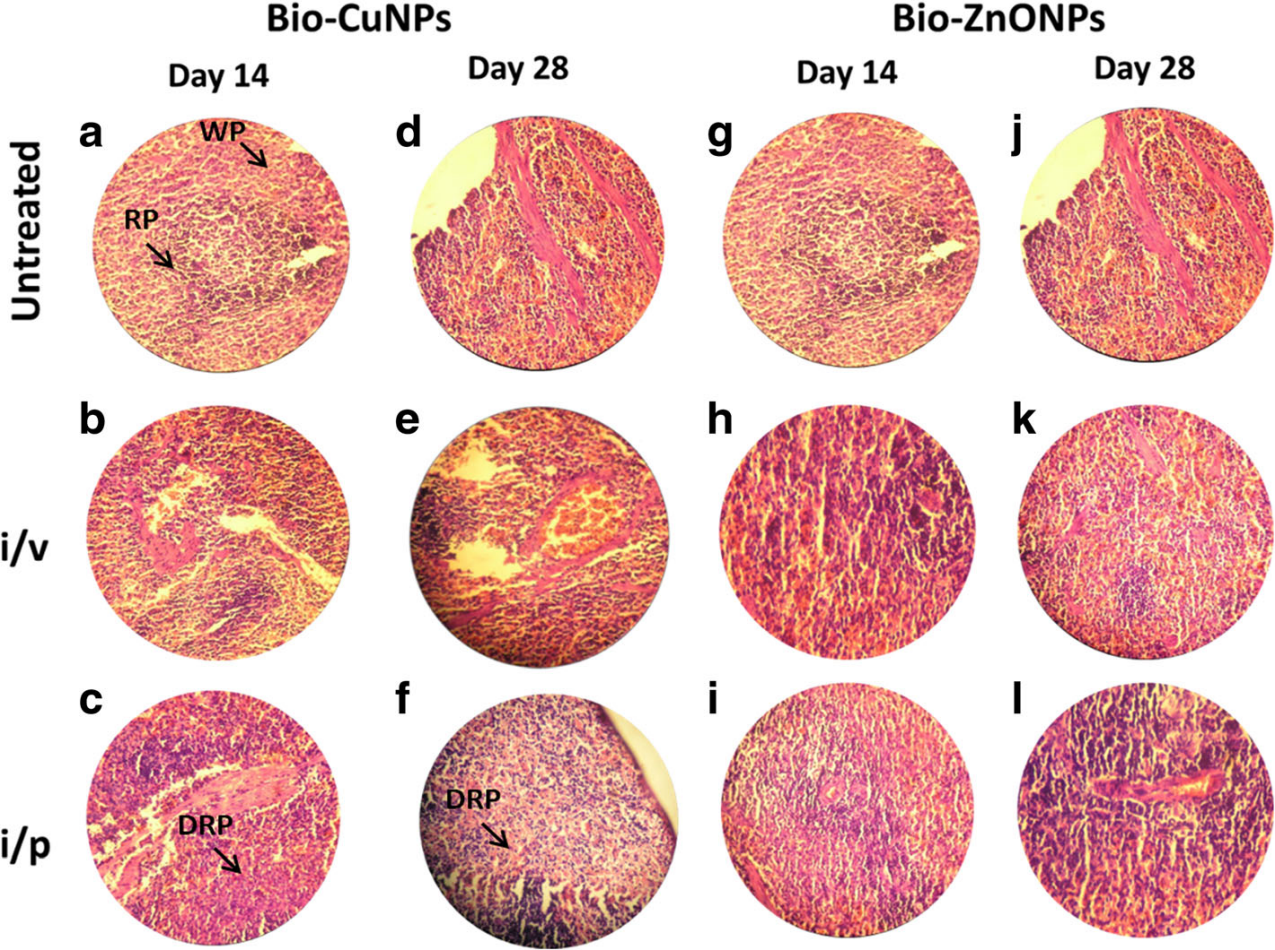
H&E-stained sections of rat spleen. Rats treated via i/v and i/p route with Bio-Cu and ZnONPs; untreated samples were considered as control. Sections treated with Bio-CuNPs observed on day 14 (A–C) and day 28 (D–F). Bio-ZnONPs’ treated sections on day 14 (G–I) and day 28 (J–L). WP white pulp, RP red pulp, DRP decline in red pulp

**Fig. 6:**
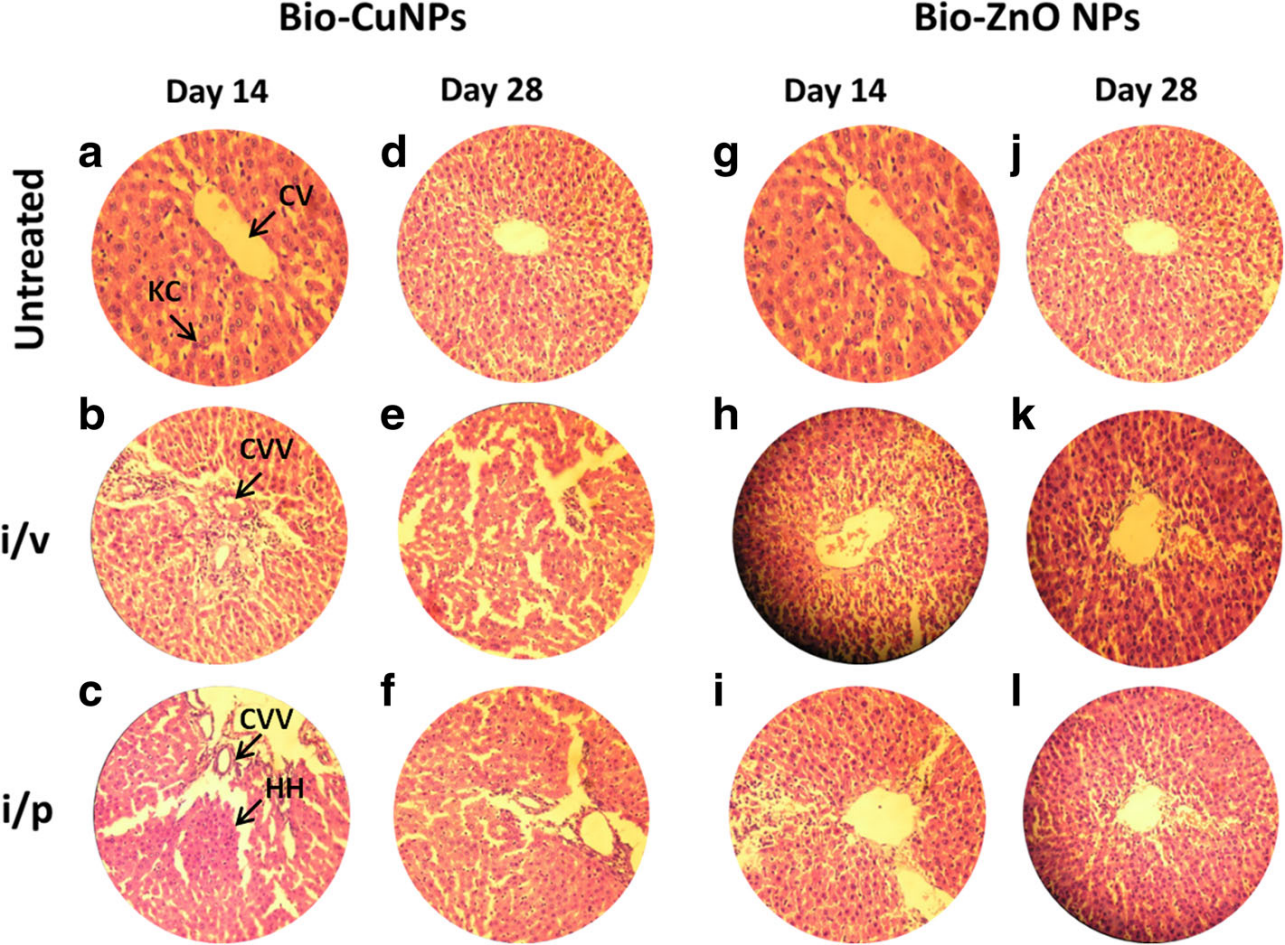
H&E-stained sections of rat liver. Rats treated via i/v and i/p route with Bio-Cu and ZnONPs; untreated samples were considered as control. Sections treated with Bio-CuNPs observed on day 14 (A–C) and day 28 (D–F). Bio-ZnONPs’ treated sections on day 14 (G–I) and day 28 (J–L). CV central vein, KC Kupffer cells, CVV central vein vacuolization (cytoplasmic vacuolization), HH hepatic haemorrhage

**Fig. 7:**
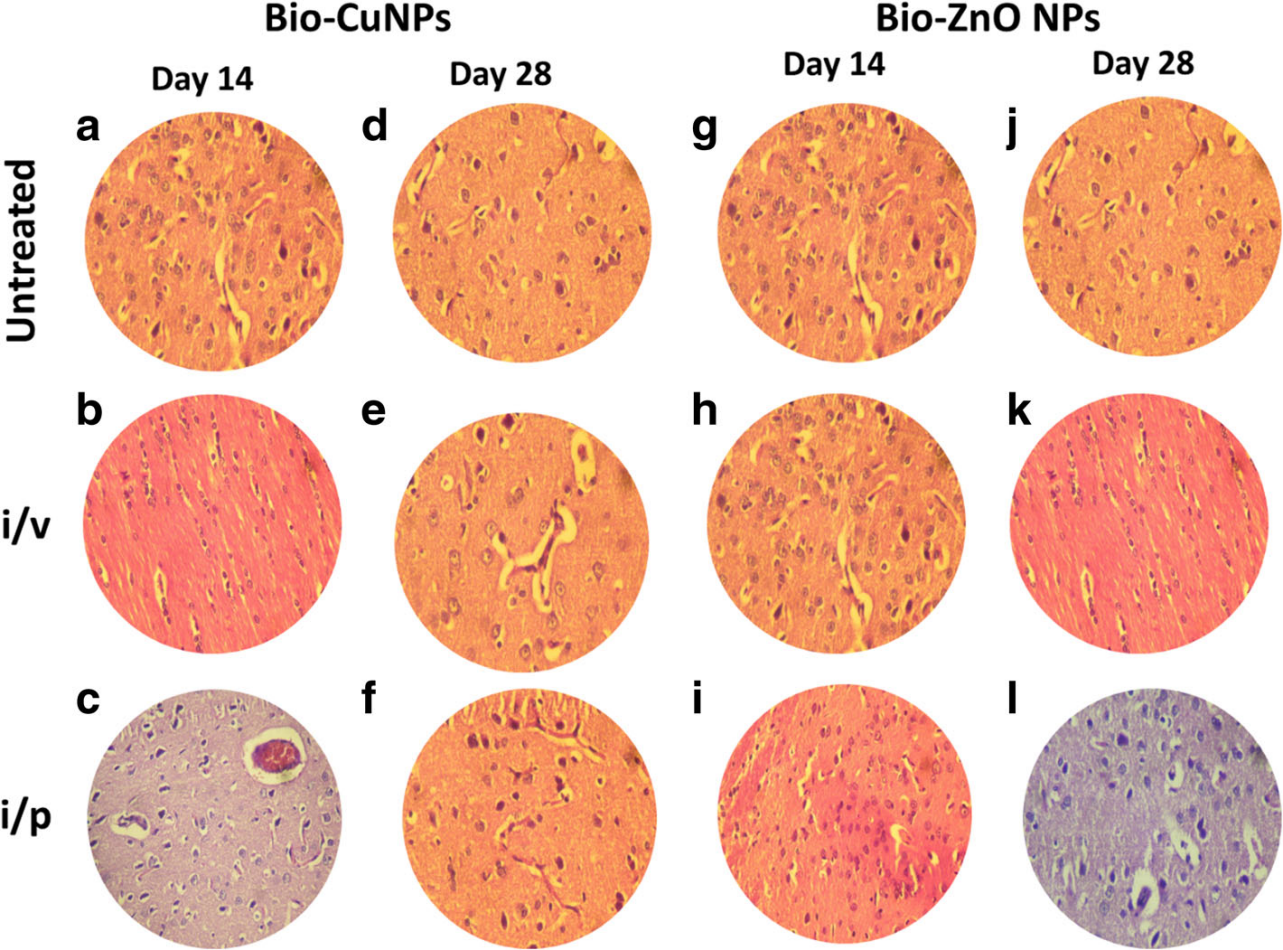
H&E-stained sections of rat brain. Rats treated via i/v and i/p route with Bio-Cu and ZnONPs; untreated samples were considered as control. Sections treated with Bio-CuNPs observed on day 14 (A–C) and day 28 (D–F). Bio-ZnONPs’ treated sections on day 14 (G–I) and day 28 (J–L). [All slides are observed under × 40 magnification, NIKON eclipse E200 (Trinacular microscope)]

We tried to scrutinize the effects of toxicity, at different doses and time intervals of Bio-NPs. Tissues treated with Bio-ZnONPs (i/v route: dosage range 11-16 μg/kg, i/p route: dosage range 24-30 μg/kg) showed no changes in the liver, kidney, spleen and brain when compared with control tissues (Figs. 4, 5, 6 and 7). Necropsy observations (autopsy: dissective examination of dead rat) specified that all organs of Bio-NPs treated rats exhibited the anatomic features (e.g., characteristics of color, shape, and size) to be expected based on their appearance in untreated animal. As compared to Bio-ZnONPs, Bio-CuNPs treated rats showed more significant changes in anatomic features of kidney, liver, spleen and brain tissues in contrast to control (Figs. 4, 5, 6 and 7).

Bio-CuNPs induced damages via i/v route administration and showed dose-dependent trend in tissues. At TLC (11. 7 μg/kg) concentration, Bio-CuNPs showed severe damage to the liver and kidney tissues of Wistar rats. In addition, Bio-CuNPs treated via both i/v and i/p route have induced necrosis of glomerular cells (glomerular atrophy), bowman capsule and proximal tubular in group 14th and 28th day rats (Fig. 4b, e, c and f) when compared to the untreated control. Glomerular necrosis is due to immunological reimbursement, but tubule damages are mostly due to toxic effect of the NPs. The tubular damage caused by the toxic effects of Bio-CuNPs through both i/v and i/p route also increased the glomerular pressure and causes glomerular atrophy.

Normal control group showed normal histological structure of hepatic lobule and the central vein which is surrounded by normal hepatocytes (Fig. 6). Bio-CuNPs treated via i/p route (19.82 μg/kg) showed mild histological changes including Kupffer cell activation both in 14th and 28th day observation (Fig. 6b, e). Rats treated with Bio-CuNPs via i/v route showed severe changes including cytoplasmic vacuolization of central vein surrounded hepatocytes and hepatic haemorrhage for 14th day observation (Fig. 6c). Surprisingly, 28th day group showed moderate changes including fatty changes of hepatocytes and pyknosis of hepatocytic nuclei (Fig. 6f). Liver tissue treated with Bio-ZnONPs, for 14th day of i/p route administered group, showed moderate changes demonstrated by fatty changes of hepatocytes (Fig. 6i). Bio-ZnONPs’ treated groups showed slight improvement, and significant hepatoprotective activity was observed in 28th day as compared to 14th day (Fig. 6l). On 14th day onwards, restoration of normal hepatic architecture has taken place in Bio-ZnONPs’ treated animals.

A decline in the spleen cell (red pulp), whereas an increase in lymphocytes (white pulp), in spleen tissue extracted from rat treated with Bio-CuNPs via i/v route (Fig. 5c, f) was noticed. In primary observation, accretion of Bio-CuNPs in the spleen was noticed in the red pulp and was related with a modest loss of cell mass; reduced cell mass was obvious on the 28th day time point when compared with 14th day of i/v administration (Fig. 5f), whereas minor changes were observed in red pulp depletion when rats were treated with Bio-CuNPs via i/p route on both 14th and 28th day time point. Structural changes were not seen in the white pulp or in splenic blood vessels (arteries or venous sinuses) and intravascular erythrocytes (Fig. 5b, e). No morphological changes have been found in spleen tissues treated with Bio-ZnONPs (Fig. 5). The H&E-stained brain sections of rats, treated (i/v and i/p) with NPs, showed no changes in brain region, olfactory bulb (perivascular localization) and the choroid plexus and ependyma of the lateral ventricles (Fig. 7).

## Conclusion

Animal toxicity studies using 16- to 96-nm-ranged biosynthesized copper (Bio-CuNPs) and zinc oxide (Bio-ZnONPs) was assessed in male Wistar rat at the dose range of 6.1 to 19.82 μg/kg and 11.14 to 30.3 μg/kg respectively for both i/p and i/v routes on 14th and 28th day of observation. We observed no mortality and normal behaviour in the animals treated with Bio-CuNPs and Bio-ZnONPs in their specific dose range. The results also verified the Bio-CuNPs and Bio-ZnONPs at low concentrations do not cause identifiable toxicity even after their breakdown in vivo over time. Increased concentrations of these Bio-NPs induce weight reduction, but no significant statistical difference was observed for Bio-ZnONPs’ treated animals. In the case of i/v and i/p Bio-CuNPs’ treated groups, obvious effects on organ index have been observed at both IC_50_ and TLC doses. Moreover, of the two different administration routes, the intraperitoneal injection shows the modest toxicity in Bio-ZnONPs’ treated groups and highest toxicity in Bio-CuNPs’ treated groups. Correspondingly, the intravenous injection shows the least toxicity in Bio-ZnONPs’ treated groups and highest toxicity in Bio-CuNPs’ treated groups. Considering all the results of studies, targeting Bio-ZnONPs by intravenous injection is promising for possible biomedical application.

## Additional file

Additional file 1: **Figure S1.** FeSEM images of Bio-CuNPs from *Enterococcus faecalis* [32]. Figure S2: FeSEM images of Bio-ZnONPs from *Enterococcus faecalis* [37]. (DOCX 1058 kb)

Additional file 2: **Figure S3**. TEM images of (A) Bio-CuNPs and (B) Bio-ZnONPs synthesized from *Enterococcus faecalis* [41]. (DOCX 901 kb)

## Abbreviations

±: Plus or minus
ALP: Alkaline phosphatase
ALT: Alanine aminotransferase
AST: Aspartate aminotransferase
Bio-CuNPs: Biogenic copper nanoparticles
Bio-ZnONPs: Biogenic zinc oxide nanoparticles
B_X_: Brain index
CRE: Creatinine
Cu: Copper
FesEM: Field emission scanning electron microscopy
H&E: Haematoxylin eosin
Hb: Haemoglobin
H_X_: Heart index
i.e.: That is
i/p route: Intraperitoneal route
i/v route: Intravenous route
IC: Inhibitory concentration
K_X_: Kidney index
Li_X_: Liver index
Lu_X_: Lung index
NOAEC: No observable adverse effect concentration
NPs: Nanoparticles
O_x_: Organ index
RBC: Red blood cell
SD: Standard deviation
S_X_: Spleen index
TEM: Transmission electron microscopy
TLC: Total lethal concentration
T_X_: Thymus index
WBC: White blood cell
ZnO: Zinc oxide

## References

[1] Etheridge ML, Campbell SA, Erdman AG, Haynes CL, Wolf SM, McCullough J (2013). The big picture on nanomedicine: the state of investigational and approved nanomedicine products. Nanomedicine.

[2] Unsal YE, Soylak M, Tuzen M, Hazer B (2015). Determination of lead, copper, and iron in cosmetics, water, soil, and food using polyhydroxybutyrate-B-polydimethyl siloxane preconcentration and flame atomic absorption spectrometry. Anal Lett.

[3] Sharma V, Singh P, Pandey AK, Dhawan A (2012). Induction of oxidative stress, DNA damage and apoptosis in mouse liver after sub-acute oral exposure to zinc oxide nanoparticles. Mutat Res.

[4] Strain JJ, Cashman KD (2009). Minerals and trace elements.Introduction to human nutrition.

[5] Iakovidis I, Delimaris I, Piperakis SM (2011). Copper and its complexes in medicine: a biochemical approach. Mol Biol Int.

[6] Wang ZL (2004). Zinc oxide nanostructures: growth, properties and applications. J Phys.

[7] Sharifi S, Behzadi S, Laurent S, Forrest ML, Stroeve P, Mahmoudi M (2012). Toxicity of nanomaterials. Chem Soc Rev.

[8] Roychoudhury S, Nath S, Massanyi P, Stawarz R, Kacaniova M, Kolesarova A (2016). Copper-induced changes in reproductive functions: in vivo and in vitro effects. Physiol Res.

[9] USEPA (United States Environment Protection Agency) (1986). Guidance for reregistration of pesticide products containing copper sulfate.

[10] Spitalny KC, Brondum J, Vogt RL, Sargent HE, Kappel S (1984). Drinking-water-induced copper intoxication in a Vermont family. Pediatrics.

[11] Solomon EI, Heppner DE, Johnston EM, Ginsbach JW, Cirera J, Qayyum M, Kieber-Emmons MT, Kjaergaard CH, Hadt RG, Tian L (2014). Copper active sites in biology. Chem Rev.

[12] Kong L, Gao X, Zhu J, Cheng K, Tang M (2016). Mechanisms involved in reproductive toxicity caused by nickel nanoparticle in female rats. Environ Toxicol.

[13] Yang Y, Qin Z, Zeng W, Yang T, Cao Y, Mei X, Kuang Y (2016) Toxicity assessment of nanoparticles in various systems and organs. Nanotechnol Rev. 6(3):279–289.

[14] Zabłocka-Słowińska K, Grajeta H (2017) Selenium and copper in type 2 diabetes mellitus-more doubt than certainty. J Elem 22(1)

[15] Zhang L, Bai R, Liu Y, Meng L, Li B, Wang L, Chen C (2012). The dose-dependent toxicological effects and potential perturbation on the neurotransmitter secretion in brain following intranasal instillation of copper nanoparticles. Nanotoxicology.

[16] Ruttkay-Nedecky B, Nejdl L, Gumulec J, Zitka O, Masarik M, Eckschlager T, Kizek R (2013). The role of metallothionein in oxidative stress. Int J Mol Sci.

[17] Mu Q, Jiang G, Chen L, Zhou H, Fourches D, Tropsha A, Yan B (2014). Chemical basis of interactions between engineered nanoparticles and biological systems. Chem Rev.

[18] Ben-Slama I, Mrad I, Rihane N, Mir LE, Sakly M, Amara S (2015). Sub-acute oral toxicity of zinc oxide nanoparticles in male rats. J Nanomed Nanotechnol.

[19] Cozzi E, Wingard CJ, Cascio WE, Devlin RB, Miles JJ, Bofferding AR, Henriksen RA (2007). Effect of ambient particulate matter exposure on hemostasis. Transl Res.

[20] Han W, Yu Y, Li N, Wang L (2011). Application and safety assessment for nano-composite materials in food packaging. Chin Sci Bull.

[21] Wang L, Wang L, Ding W, Zhang F (2010). Acute toxicity of ferric oxide and zinc oxide nanoparticles in rats. J Nanosci Nanotechnol.

[22] Zhang H, Ji Z, Xia T, Meng H, Low-Kam C, Liu R, Pokhrel S, Lin S, Wang X, Liao YP, Wang M (2012). Use of metal oxide nanoparticle band gap to develop a predictive paradigm for oxidative stress and acute pulmonary inflammation. ACS Nano.

[23] Bai X, Li L, Liu H, Tan L, Liu T, Meng X (2015). Solvothermal synthesis of ZnO nanoparticles and anti-infection application in vivo. ACS Appl Mater Interfaces.

[24] Kim JS, Adamcakova-Dodd A, O'Shaughnessy PT, Grassian VH, Thorne PS (2011). Effects of copper nanoparticle exposure on host defense in a murine pulmonary infection model. Part Fibre Toxicol.

[25] Saptarshi SR, Feltis BN, Wright PF, Lopata AL (2015). Investigating the immunomodulatory nature of zinc oxide nanoparticles at sub-cytotoxic levels in vitro and after intranasal instillation in vivo. J Nanobiotechnol.

[26] Liao M, Liu H (2012). Gene expression profiling of nephrotoxicity from copper nanoparticles in rats after repeated oral administration. Environ Toxicol Pharmacol.

[27] Esmaeillou M, Moharamnejad M, Hsankhani R, Tehrani AA, Maadi H (2013). Toxicity of ZnO nanoparticles in healthy adult mice. Environ Toxicol Pharmacol.

[28] Jacobsen NR, Stoeger T, Van Den Brûle S, Saber AT, Beyerle A, Vietti G, Banerjee A (2015). Acute and subacute pulmonary toxicity and mortality in mice after intratracheal instillation of ZnO nanoparticles in three laboratories. Food Chem Toxicol.

[29] Prabhu BM, Ali SF, Murdock RC, Hussain SM, Srivatsan M (2010). Copper nanoparticles exert size and concentration dependent toxicity on somatosensory neurons of rat. Nanotoxicology.

[30] Smijs TG, Bouwstra JA (2010). Focus on skin as a possible port of entry for solid nanoparticles and the toxicological impact. J Biomed Nanotechnol.

[31] Ashajyothi C, Jahanara K, Kelmani Chandrakanth R (2014). Biosynthesis and characterization of copper nanoparticles from Enterococcus faecalis. Int J Pharm Biosci.

[32] Ashajyothi C, Manjunath R, Narasanna K, Chandrakanth R (2014). Antibacterial activity of biogenic zinc oxide nanopaticals synthesized from enterococcus faecalis. Int J ChemTech Res.

[33] Zhang XD, Wu HY, Wu D, Wang YY, Chang JH, Zhai ZB, Meng AM, Liu PX, Zhang LA, Fan FY (2010). Toxicologic effects of gold nanoparticles in vivo by different administration routes. Int J Nanomedicine.

[34] Hong TK, Tripathy N, Son HJ, Ha KT, Jeong HS, Hahn YB (2013). A comprehensive in vitro and in vivo study of ZnO nanoparticles toxicity. J Mater Chem B.

[35] Bergin IL, Witzmann FA (2013). Nanoparticle toxicity by the gastrointestinal route: evidence and knowledge gaps. Int J Biomed Nanosci Nanotechnol.

[36] Sarhan OM, Hussein RM (2014). Effects of intraperitoneally injected silver nanoparticles on histological structures and blood parameters in the albino rat. Int J Nanomedicine.

[37] ZiaeeGhahnavieh M, Ajdary M, ZiaeeGhahnavieh M, Naghsh N (2014). Effects of intraperitoneal injection of gold nanoparticles in male mice. Nanomed J.

[38] De Jong WH, Van Der Ven LT, Sleijffers A, Park MV, Jansen EH, Van Loveren H, Vandebriel RJ (2013). Systemic and immunotoxicity of silver nanoparticles in an intravenous 28 days repeated dose toxicity study in rats. Biomaterials.

[39] Rhiouani H, El-Hilaly J, Israili ZH, Lyoussi B (2008). Acute and sub-chronic toxicity of an aqueous extract of the leaves of Herniariaglabra in rodents. J Ethnopharmacol.

[40] Kim D, Park S, Lee JH, Jeong YY, Jon S (2007). Antibiofouling polymer-coated gold nanoparticles as a contrast agent for in vivo X-ray computed tomography imaging. J Am Chem Soc.

[41] Ashajyothi C, Handral HK, Dubey N, Kelmani Chandrakanth R (2016) Antibiofilm activity of biogenic copper and zinc oxide nanoparticles-antimicrobials collegiate against multiple drug resistant bacteria: a nanoscale approach. J Nanostruct Chem (4):329–341

