# Supplementary material for: A Comparative In Vivo Scrutiny of Biosynthesized Copper and Zinc Oxide Nanoparticles by Intraperitoneal and Intravenous Administration Routes in Rats
Source: Nanoscale Res Lett. 2018 Apr 3;13:93. doi: 10.1186/s11671-018-2497-2 (PMC5882480; doi:10.1186/s11671-018-2497-2)
Supplement: Supplementary file 2 — Figure S3. TEM images of (A) Bio-CuNPs and (B) Bio-ZnONPs synthesized from Enterococcus faecalis [41]. (DOCX 901 kb) [file 11671_2018_2497_MOESM2_ESM.docx]

**A comparative *in vivo* scrutiny of Biosynthesized Copper and Zinc oxide nanoparticles by Intraperitoneal and Intravenous administration routes in rats**

C. Ashajyothi ^1^, Handral Harish K ^2^ and R. Kelmani Chandrakanth^1*^

#
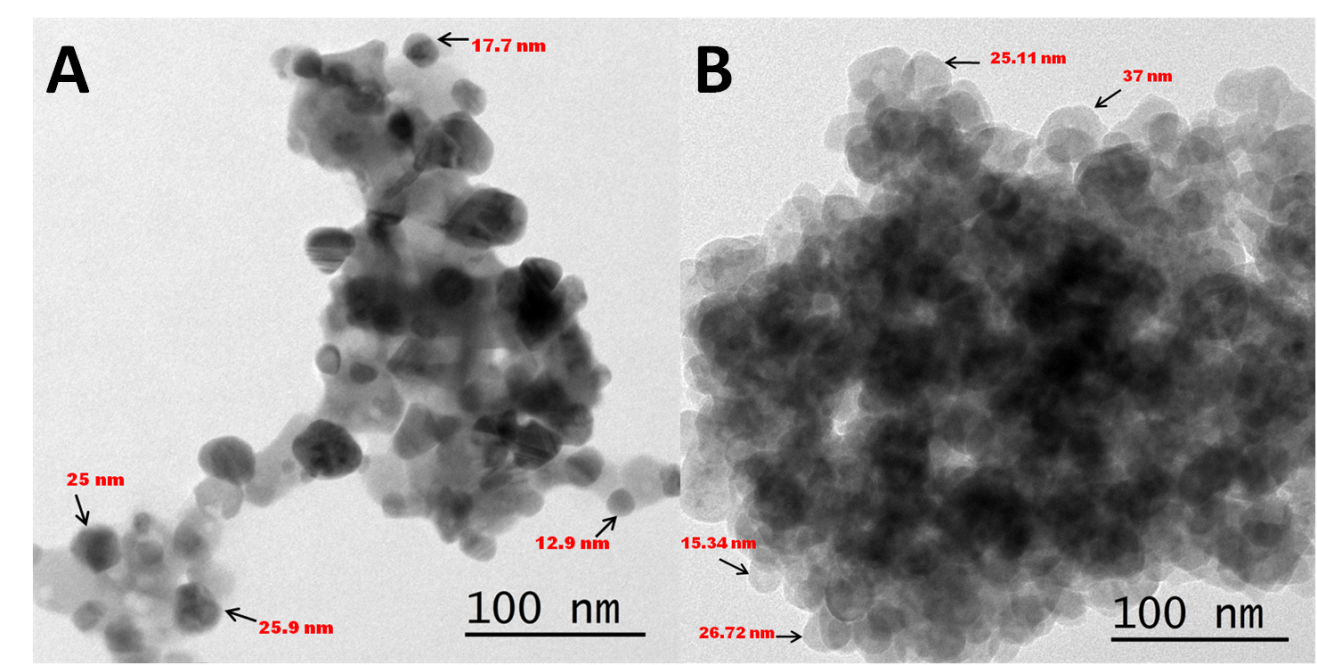


Figure: TEM images of **(A)** Bio-CuNPs and **(B)** Bio-ZnONPs synthesized from *Enterococcus faecalis* (Ashajyothi *et al.,* 2016)
